# Isolation, identification, and drug resistance of a partially isolated bacterium from the gill of *Siniperca chuatsi*

**DOI:** 10.1515/biol-2022-0608

**Published:** 2023-06-06

**Authors:** Zheng-Min Qian, Xiao-Shu Xu, Chun-Tao Li, Pei-Yong Song, Qing-Rong Wang

**Affiliations:** Biological and Agricultural Science Technology Institute, Zunyi Normal College, Zunyi, Guizhou, 563006, China

**Keywords:** *Siniperca chuatsi*, molecular identification, 16S rRNA, achromatic bacilli, drug resistance

## Abstract

This study was envisaged to identify a strain of bacteria isolated from the gill of mandarin fish. Identification and characterization of the bacterial strain were performed using morphological characteristics, growth temperature, physiological and biochemical tests, antibiotic sensitivity tests, artificial infection tests, and 16S rRNA gene sequencing homology analysis. The results showed that the bacterium was Gram-negative, with flagella at the end and the side. The bacterium exhibited a light brownish-gray colony on the Luria-Bertani culture and white colony on the blood agar plate without hemolytic ring. Normal growth was achieved at 42°C, and growth could be delayed in 7% NaCl broth medium. By homology comparison and analysis, the phylogenetic tree was constructed using MEGA7.0, and the bacterium was preliminarily identified as *Achromobacter*. The antibiotic sensitivity test showed that the strain was sensitive to piperacillin, carbenicillin, cefoperazone, cefazolin, ofloxacin, gentamicin, kanamycin, amikacin, neomycin, erythromycin, minocycline, doxycycline, polymyxin B, tetracycline, chloramphenicol, and other drugs. However, it was resistant to penicillin, ampicillin, oxacillin, ceftriaxone, cefradine, cefalexin, cefuroxime sodium, ciprofloxacin, norfloxacin, vancomycin, compound sulfamethoxazole, clindamycin, medimycin, and furazolidone.

## 1 Introduction

*Siniperca chuatsi* is one of the famous freshwater fish in China. It has more meat and fewer bones, and its meat is fresh and tender. It is considered to be a treasure of fish and a precious fish that is popular among the consumers. Since the early 1970s, China has started the research on the artificial propagation of mandarin fish and has developed the techniques of artificial propagation, seedling cultivation, and pond culture of adult fish. In the high-density intensive aquaculture water body, the pollution sources are not only the external inputs such as industrial wastewater and agricultural surface resources pollution, but also the endogenous inputs such as residual bait, metabolites, and medicines. These sources lead to illness and even death of farmed animals, thereby seriously affecting the water quality and safety of the aquatic products. In recent years, with the expansion of the aquaculture scale and aquaculture density, water pollution remains to be a serious issue, which brings huge economic losses and serious disasters to aquaculture production, hindering the development of aquaculture [1].

**Figure 1:**
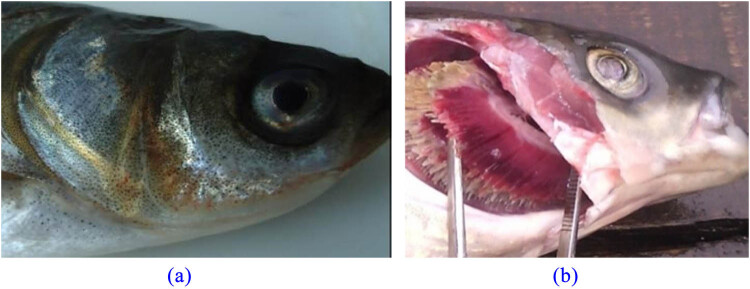
Gill disease of *S. chuatsi.* Note: (a) as surface morphology of gill cap, (b) as gill filament morphology.

With the aggravation of water pollution, the diseases of cultured fish are becoming more and more serious. Fish gill rot caused by bacteria is a common fish disease. When seen with the naked eye, the diseased fish looks blackened, with the blackest head, commonly known as aconite plague (Figure 1a). In the fish, the disease manifests with slow swimming, slow response to external stimuli, dyspnea, and loss of appetite. When the disease is serious, the fish swims alone and does not eat. When the gill cover of such a diseased fish is observed, it can be seen that the gill line of the diseased fish is yellow. The gill line decays with sludge, especially at the end of the gill line. There is a lot of mucus, which often sticks to sludge and debris. Sometimes, blood spots can also be seen on the gill flap (Figure 1b). The inner epidermis of the operculum is often congested. In this study, the samples were isolated from the gills of mandarin fish with obvious symptoms of gill rot disease, and morphological observation, artificial infection test, molecular identification, antibiotic sensitivity test, etc., were carried out, in order to provide a theoretical basis for the cultivation of mandarin fish and the prevention and control of bacterial diseases.

## 2 Materials and methods

### 2.1 Isolation and purification of suspicious pathogenic bacteria

Diseased samples of *S. chuatsi* were collected from the Wujiangdu reservoir farm in April 2021. After being packaged in sterile food bags, they were placed in refrigerated boxes and brought back to the laboratory. The bacteria were immediately isolated on the ultra-clean workbench.

The gill lids of *S. chuatsi* with obvious symptoms of gill rot were cut off; the gill filaments were sampled with sterilized cotton swabs and coated on Luria-Bertani (LB) plates; the plates were taken out; different colonies on the plates were separated by the method of line dilution according to the colony morphology; and the colonies were transferred to blank LB culture. One colony was transferred to one plate, and the plate was inverted in 30°C incubator, and the aforementioned steps were repeated until the colonies grew on the same plate and were identified as the same colony in morphology.

### 2.2 Morphological characteristics

A single colony was selected and cultured in LB liquid medium at 30°C for 12–16 h. The bacteria were smeared on LB and blood agar plates to observe the colony morphology, Gram staining, and microscopic examination. A single colony was selected and inoculated on the newly prepared LB slant medium (the bottom of the slant was added with a small amount of condensate) and cultured for 12–16 h, and then, the flagella staining and microscopic examination were carried out. These methods were based on the manual for *systematic identification of common bacteria compiled* by Dong et al. [2].

### 2.3 Growth and alkali-producing characteristics of the strain

The strain was inoculated into the carbonate-mineralized bacteria screening medium and sampled every 3 h (under aseptic conditions) for the determination of optical density at 600 nm, in order to characterize the growth of the strain. At the same time, another part of the sample was used to determine the pH value using a pH meter, and the experimental data were processed by MS Excel software.

### 2.4 Physiological and biochemical identification

The isolated strains were identified by biochemical tests for oxidase, contact enzyme, acid and gas production from glucose, sugar fermentation, hydrogen sulfide, nitrate reduction and citrate utilization, etc. The identification method was based on the manual for *systematic identification of common bacteria compiled* by Dong et al. [2].

### 2.5 Artificial infection test

The isolates were inoculated on LB plate and cultured at 30°C for 12–16 h. Moss was washed with aseptic saline, and the suspension was diluted to reach a concentration of 1.0 × 10^6^, 1.0 × 10^7^, 1.0 × 10^8^, and 1.0 × 10^9^ colony-forming units (CFUs)/mL. Fifty healthy *S. chuatsi* fishes were randomly divided into four test groups and one control group, with ten fish in each group. Each group was injected with 0.2 mL of bacteria solution at the base of dorsal fin, and the control group was injected with 0.2 mL of normal saline. The animals were fed in 34 × 21 × 25 cm glass tank. The water used for breeding was deflated 1 day ahead of time, and the water was changed at regular intervals every day. The animals were continuously oxygenated and observed for 7 days, and the morbidity and mortality were recorded.

### 2.6 Molecular identification

Bacterial DNA was extracted according to the method described in the study by Wei [3]. About 1.5 mL of bacteria solution was cultured at 37°C for 12–14 h in 2.0 mL eppendorf (EP) tube and centrifuged at 4,000 rpm for 1 min, and the supernatant was discarded to collect the bacteria. Subsequently, 650 μL of Tris–ethylenediamine tetraacetic acid buffer solution, 5 μL of proteinase K (20 mg/mL), and 5 μL of lysozyme (20 mg/mL) were added, respectively, in the EP tubes. The mixture was hold at 37°C for 0.5 h and then centrifuged at 12,000 rpm for 5 min. The supernatant was transferred to another 2.0 mL EP tube, and the same volume of chloroform–isoamyl alcohol was added and mixed and centrifuged at 12,000 rpm for 5 min. The supernatant was transferred to another EP tube containing 1/5 volume of sodium acetate (5 moles per liter, M) and double volume of anhydrous ethanol (precooled at −20°C), mixed well, and centrifuged at 12,000 rpm for 5 min. The ethanol was discarded, and the DNA was washed with 1 mL of 70% ethanol; the supernatant was removed and dried in air; 100 μLTE buffer solution was added to completely dissolve the DNA. The DNA samples were stored at −20°C for later use.

Universal primers (27F and 1542R) [4], 27F:5′-gagtttgatcctggctc-3′ and 1542R:5′ -agaaaggtgatccagc-3′, were used to amplify 16S rRNA using the Bio-Rad S1000 gradient polymerase chain reaction (PCR) instrument. The amplification system was as follows: 2xTaq PCR Mix (Shanghai Shenggong) 25 mL of primers 1 μL each, 1 μL of DNA, supplemented with ddH_2_O to 50 μL. Amplification procedure was as follows: predenaturation at 95°C for 3 min, denaturation at 95°C for 45 s, annealing at 55°C for 45 s, extension at 72°C for 45 s, for a total of 35 cycles, and finally extension at 72°C for 5 min. The amplified products were detected by 1% agarose gel electrophoresis and sent to Bioengineering (Shanghai) Co., Ltd., for sequencing. The sequencing data were entered into the NCBI database (http: www.NCBI.nlm.Nih.gov), the 16SrRNA gene sequences of 12 similar bacteria were downloaded from GenBank and compared using the BLAST tool, and the phylogenetic tree was constructed using Mega7.0 software.

### 2.7 Antibiotic sensitivity test

According to the method described in the study by Jia et al. [5], the concentration of the bacterial solution was adjusted to 1.0 × 10^8^ CFU/mL by means of McIntosh turbidimetric method, and the plate was evenly coated in the super-clean workbench. Antimicrobial susceptibility test of 30 antimicrobial agents (purchased from Hangzhou Tianhe Microbial Reagent Co., Ltd.) was performed, the size of the antimicrobial circle was measured, and the results of the antimicrobial susceptibility test were recorded, with reference to Hangzhou Tianhe Microbial Reagent Co., Ltd., “Antimicrobial susceptibility test paper method interpretation of the scope of the standard” to judge the bacteria to different drug sensitivity.

## 3 Results

### 3.1 Morphological characteristics of bacteria

**Figure 2:**
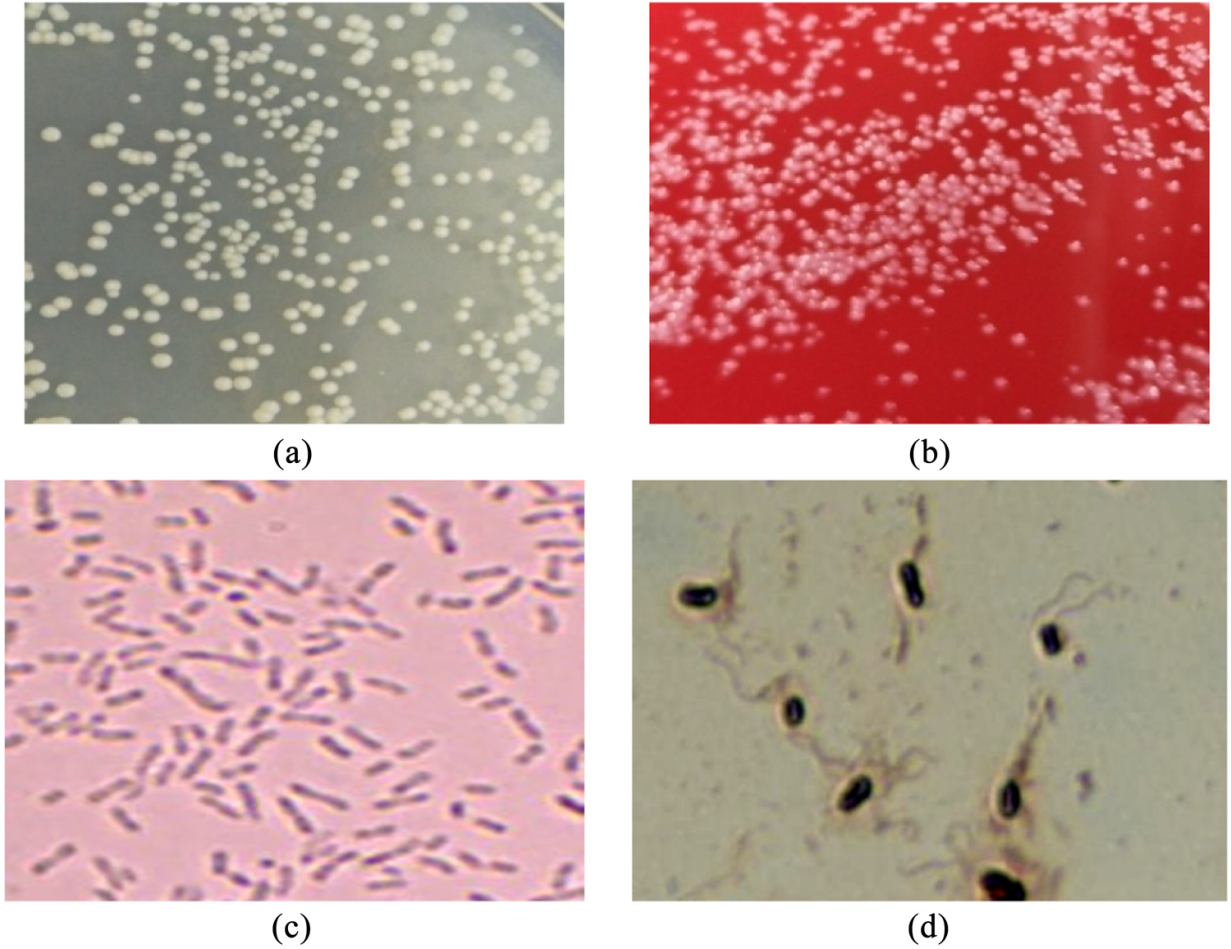
Morphological characteristics of bacteria. Note: (a) as LB medium, (b) as blood agar medium, (c) as Gram staining (1,000×), and (d) as flagellar staining (1,000×).

On the LB plate, the bacteria exhibited a round, smooth, moist, slightly raised, semitransparent, and light brown-gray colony (Figure 2a). On the blood agar plate, there was the presence of a round, smooth, moist, slightly raised white colony with an opaque edge and no hemolytic ring (Figure 2b). Gram staining showed a round colony at both ends, most of which were arranged in tandem, forming long chains in the liquid medium (Figure 2c). Microscopic examination of flagella staining revealed that the flagella were terminal lateral flagella (Figure 2d).

### 3.2 Growth and alkali-producing characteristics of the strain

**Figure 3:**
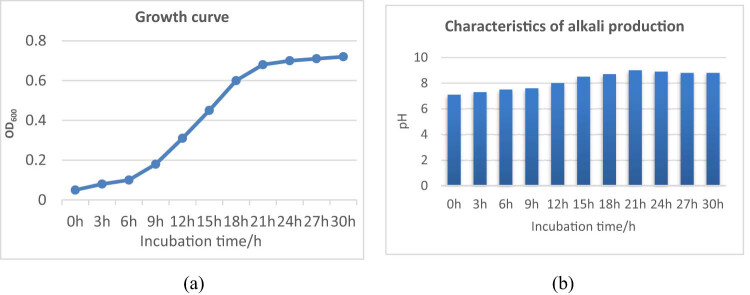
Growth and alkali production characteristics of the strain. Note: (a) as growth curve, (b) as characteristics of alkali production.

The strain was inoculated into the carbonate-mineralized bacteria-screening medium to study its growth and alkali-producing characteristics. The strain grew rapidly in 12–18 h, a logarithmic growth period, and then grew slowly into a stable period after 21 h (Figure 3a). The pH of the fermentation broth gradually increased with the increase in culture time, reaching 9.05 after 21 h, and then tended to stabilize (Figure 3b).

### 3.3 Physiological and biochemical characteristics

Biochemical experiments were performed using oxidase, contact enzyme, acid and gas production from glucose, sugar fermentation, hydrogen sulfide, nitrate reduction, and citrate utilization. Biochemical experiments results showed that the oxidase, contact enzyme, nitrate reduction, nitrite reduction, hydrogen sulfide test, *o*-nitrophenyl-β-d-galactopyranoside (ONPG), lysine hydrolase, ornithine hydrolase, and citrate activities were positive, while the acid production by glucose fermentation, mannitol fermentation, xylose fermentation, fructose fermentation, sucrose fermentation, starch hydrolysis, gelatin liquefaction, arginine double hydrolysis, and urease test was negative. Meanwhile, the bacteria could grow normally at 42°C, and the growth was delayed in 7% NaCl broth medium (the medium became cloudy after72 h).

### 3.4 Artificial infection test

Healthy *S. chuatsi* fish were injected with 1.0 × 10^6^, 1.0 × 10^7^, 1.0 × 10^8^, and 1.0 × 10^9^ CFU/mL bacteria, respectively, and the control group was injected with normal saline. The incidence and mortality were recorded after continuous oxygenation for 7 days. After 7 days of observation, no disease was found in *S. chuatsi* injected with the bacteria and saline, which indicated that the strain was not pathogenic to *S. chuatsi* or did not cause the disease alone.

### 3.5 Molecular identification

**Figure 4:**
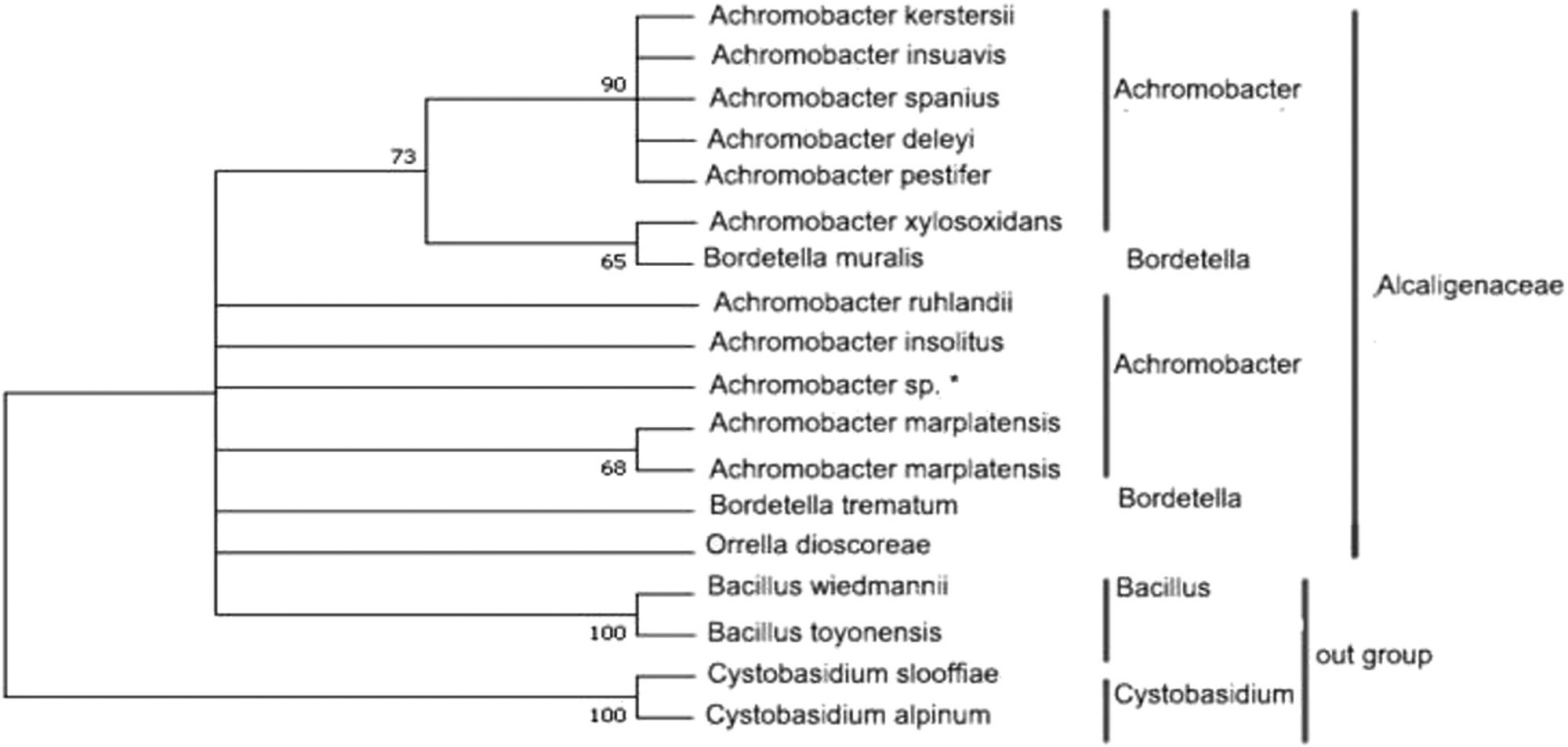
Phylogenetic tree based on 16S rRNA construction. Note: *Is this strain.

The 16S rRNA gene sequence was submitted to GenBank database with accession number MZ-734483, as shown in Table 2. The sequence was entered into NCBI website, and the homology of the sequence was analyzed by BLAST tool. The phylogenetic tree was constructed by Mega7.0, and the results are shown in Table 2 and Figure 4.

### 3.6 Antibiotic sensitivity test

The susceptibility test of 30 antimicrobial agents in common use in farm was carried out, and the results showed that the bacterium was sensitive to piperacillin, carbenicillin, cefoperazone, cefazolin, ofloxacin, gentamicin, kanamycin, amikacin, neomycin, erythromycin, minocycline, doxycycline, chloramphenicol, and polymyxin b; and resistant to penicillin, ampicillin, oxacillin, ceftriaxone, cefradine, cefalexin, cefuroxime, ceftazidime, ciprofloxacin, norfloxacin, vancomycin, trimethoprim/sulfamethoxazole, clindamycin, midecamycin, tetracycline, and furazolidone, as shown in Table 3.

## 4 Discussion and conclusion

It is reported that the colony of achromatous bacilli presents a dry round shape with slight bulge, moist, light yellow, translucent, smooth, and neat edges, negative Gram staining, belonging to facultative anaerobic large bacilli, with a size of 0.8–2.0 μm × 2.0–10.0 μm. The optimum growth temperature is 25–37°C. It grows slowly or does not grow at 42°C. It is unable to ferment most sugars to produce acid and gas. Even at 25°C, the ability to ferment glucose to produce gas is extremely weak [19,20]. In this study, after the bacterial strain was cultured on the agar medium for 12 h, it was observed that the colony was transparent, light brown gray; the medium was transparent; the color of the colony did not change; the edge was smooth, slightly raised; and Gram staining was negative, as shown in Figure 2. It could reach 2 mm in 24 h on LB solid medium containing ampicillin. It belonged to the facultative anaerobic bacterial class. It was found to be parasitic on the gills of *S. chuatsi* and could grow at 20–45°C. It could not ferment most sugars to produce acid and gas. The strain was inoculated into the screening medium of carbonate-mineralizing bacteria to study its growth and alkali production characteristics. The strain grew rapidly in 12–18 h, which was a logarithmic growth period. After 21 h, it grew slowly and entered a stable period (Figure 3a). The pH value of fermentation broth gradually increased with the increase in culture time, reaching 9.05 after 21 h, and then became stable (Figure 3b). Through morphological, physiological, and biochemical experiments, this strain was found to be consistent with *Achromobacter* in morphological, physiological, and biochemical characteristics, as shown in Table 1. Through the artificial infection test, the strain exhibited no pathogenicity to *S. chuatsi*, or did not cause any disease alone. The collection site shows that the disease may be caused by other pathogens, which needs further research.

**Table 1:** Morphological and biochemical characteristics of strain

Item	Result	Item	Result
Oxidase	+	Mannitol	−
Contact enzyme	+	Xylose	−
Motility	+	Fructose	−
Gram stain	−	Sucrose	−
Glucose acid production	−	Starch hydrolysis	−
Urine enzyme	−	Gelatin liquefaction	−
Nitrate reduction	+	Arginine hydrolase	−
Nitrite reduction	+	Lysine hydrolase	+
Hydrogen sulfide	+	Ornithine hydrolase	+
ONPG	+	Citrate utilization	+
Growth at 25°C	+	Growth at 1% NaCl broth	+
Growth at 35°C	+	Growth at 3% NaCl broth	+
Growth at 42°C	+	Growth at 7% NaCl broth	+ and (delayed growth)

The 16S rRNA gene sequence of the strain was submitted to the GenBank database, and the sequence was compared and analyzed using BLAST tools. The homology between the 16S rRNA gene sequence of the strain and *Acrobat marplatensis* was up to 99.24%, and the homology with other reported *Acrobat* bacteria was more than 97%, as shown in Table 2. The phylogenetic tree was constructed by MEGA7.0, and the strain and the acrobat were clustered into one branch, which showed that it was closely related to the genus *Achromobacter*, as shown in Figure 4. Combined with its morphological, physiological, biochemical, and growth characteristics, it was confirmed that the bacterium belonged to genus *Achromobacter*.

**Table 2:** Information of 16S rRNA gene of *Achromobacter* used in this study

Similar strains	GenBank accession No.	Homology (%)	Sources
*Achromobacter deleyi*	NR_152014	98.97	Vandamme et al. [6]
*Achromobacter kerstersii*	NR_152015	98.88	Vandamme et al. [6]
*Achromobacter pestifer*	NR_152016	98.41	Vandamme et al. [6]
*Achromobacter spanius*	NR_025686	98.97	Coenye et al. [7]
*Achromobacter insolitus*	NR_025685	98.41	Coenye et al. [7]
*Achromobacter marplatensis*	NR_116198	99.24	Gomila et al. [8]
*Achromobacter insuavis*	NR_117706	98.60	Vandamme et al. [9]
*Achromobacter xylosoxidans*	NR_118403	98.23	Ridderberg et al. [10]
*Achromobacter ruhlandii*	NR_027197	97	Yabuuchi et al. [11]
*Achromobacter* sp.	**MZ-734483**		**Present study**
*Bordetella muralis*	NR_145920	96.54	Tazato et al. [12]
*Bordetella trematum*	NR_025404	95.98	von Wintzingerode et al. [13]
*Orrella dioscoreae*	NR_160523		Carlier et al. [14]
*Bacillus wiedmannii*	NR_152692		Miller et al. [15]
*Bacillus toyonensis*	NR_121761		Jimenez et al. [16]
*Cystobasidium slooffiae*	NR_103568		Scorzetti et al. [17]
*Cystobasidium alpinum*	NR_159815		Turchetti et al. [18]

From the antibiotic sensitivity test (Table 3), the bacterium was found to be resistant to penicillin, ampicillin, and oxacillin. It was resistant to ceftriaxone, cefradine, cephalexin, and cefuroxime sodium of cephalosporins. It was resistant to ciprofloxacin and norfloxacin of quinolones; vancomycin of glycopeptides; compound sulfamethoxazole of sulfonamides; clindamycin of lincosamides; midecamycin of macrolides; furazolidone of nitrofurans, etc. On the other hand, it was sensitive to piperacillin and carbenicillin of penicillins; cefoperazone of cephalosporins; ceftazidime and cefazolin of quinolones; ofloxacin of macrolides; tetracycline, minocycline, and doxycycline of tetracyclines; polymyxin B of polypeptides; gentamicin of aminoglycosides; kanamycin, amikacin, and neomycin of macrolides; and chloramphenicol.

**Table 3:** Results of antibiotic sensitivity test

Antibiotics classes	Name of antibiotic	Drug content (μg/tablet)	Criterion of inhibition zone diameter (mm)			Experimental bacteriostatic zone (mm)	Drug resistance
			R	I	S		
Penicillins	Penicillin	10	≤15	16–20	≥21	0	R
	Ampicillin	10	≤13	14–16	≥17	0	R
	Piperacillin	100	≤17	18–20	≥21	28 ± 1.5	S
	Carboxybenzyl penicillin	100	≤15	16–20	≥21	27 ± 1.7	S
	Oxacillin	10	≤15	16–20	≥21	27 ± 1.5	R
Cephalosporins	Ceftriaxone	30	≤15	16–20	≥21	0	R
	Cefradine	30	≤15	16–20	≥21	5 ± 1.2	R
	Cephalexin	30	≤15	16–20	≥21	0	R
	Cefuroxime sodium	30	≤14	15–17	≥18	0	R
	Cefoperazone	75	≤15	16–20	≥21	28 ± 1.8	S
	Cefazolin	30	≤14	15–17	≥18	20 ± 1.5	S
	Ceftazidime	30	≤14	15–17	≥18	5 ± 1.3	R
Quinolones	Ciprofloxacin	5	≤15	16–20	≥21	0	R
	Norfloxacin	10	≤12	13–16	≥17	0	R
	Ofloxacin	5	≤12	13–15	≥16	25 ± 1.5	S
Glycopeptides	Vancomycin	30	≤15	16–20	≥21	0	R
Sulfonamides	Compound sulfamethoxazole	1.25	≤10	11–15	≥16	0	R
Lincosamides	Clindamycin	2	≤15	16–20	≥21	0	R
Aminoglycosides	Gentamicin	10	≤12	13–14	≥15	30 ± 1.8	S
	Kanamycin	30	≤15	16–20	≥21	25 ± 1.7	S
	Amikacin	30	≤15	16–20	≥21	21 ± 1.8	S
	Neomycin	30	≤15	16–20	≥21	22 ± 1.7	S
Macrolides	Medimycin	30	≤15	16–20	≥21	0	R
	Erythromycin	15	≤15	16–20	≥21	21 ± 1.7	S
Tetracyclines	Tetracycline	30	≤15	16–20	≥21	32 ± 1.5	S
	Minocycline	30	≤15	16–20	≥21	35 ± 1.8	S
	Doxycycline	30	≤15	16–20	≥21	36 ± 1.9	S
Chloramphenicols	Chloramphenicol	30	≤12	13–17	≥18	40 ± 1.5	S
Polypeptides	Polymyxin B	300	≤15	16–20	≥21	27 ± 1.5	S
Nitrofuran	Furazolidone	300	≤15	16–20	≥21	0	R

Note: S: sensitivity; I: intermediate; R: resistance.

There are many reports on analysis of drug resistance of the colorless bacilli. Li et al. [21] analyzed the drug resistance of a strain of colorless bacilli collected from cucumber and found that the strain was resistant to kanamycin, chloramphenicol, ampicillin, etc., and sensitive to tetracycline. Li et al. [22] analyzed the drug resistance of a strain of achromatous bacteria from the rumen of yaks and found that the bacteria were sensitive to most of the 14 kinds of antibiotics (including ciprofloxacin), and only resistant to cephalexin and aztreonam, with low safety risk.

Wang et al. [23] analyzed the drug resistance of *Achromobacter xylosoxidans* isolated from neonatal sepsis and found that it was sensitive to compound sulfamethoxazole and resistant to gentamicin. Bador et al. [24] believed that *Achromobacter xylosoxidans* was inherently resistant to gentamycin, ofloxacin, and kanamycin. Amoureux et al. [25] believed that *Achromobacter xylosoxidans* was susceptible to acquired drug resistance to ceftazidime, ciprofloxacin, etc. Turel et al. [26] believed that piperacillin, piperacillin/tazobactam, carbapenems, and compound sulfamethoxazole were the first-choice drugs for the treatment of this bacterium. The combination of piperacillin plus gentamicin, azithromycin plus doxycycline, and azithromycin plus compound sulfamethoxazole could also achieve gratifying effects. The strains that were isolated in this study were from the gills of cultured *S. chuatsi*, and belonged to the same genus of *Achromobacter*, but sensitive to kanamycin, gentamycin, ofloxacin, chloramphenicol, and other antibiotics. On the other hand, it was resistant to ciprofloxacin, compound sulfamethoxazole, and other antibiotics (Table 3). This shows that the resistance of bacteria to antibiotics may be related to the source of bacteria and is greatly affected by the living environment.

The strain was isolated from the gills of cultured *S. chuatsi* and showed resistance to a variety of antibiotics, which may be due to the environmental pressure caused by the abuse of antibacterial drugs in the process of cultivation. Therefore, real-time monitoring of the drug resistance and its change in trend in the bacteria isolated from fish breeding sites should be periodically performed. Further, a rational, scientific, and standardized drug use should be made, in order to control and reduce the production of drug-resistant strains. These measures can offer a certain guiding significance for the resource management of fisheries and can assist in the prevention and control of the bacterial diseases in freshwater fish.

In conclusion, the bacteria were preliminarily identified that the bacterium belonged to the genus *Achromobacter* and was resistant to many commonly used antibiotics.
